# Awareness of Patients and Relatives about Breast Cancer in Turkey

**Published:** 2018-01

**Authors:** Serdar Gökay TERZİOĞLU, Murat Özgür KILIÇ, Gül DAĞLAR

**Affiliations:** Dept. of General Surgery, Numune Training and Research Hospital, Ankara, Turkey

## Dear Editor-in-Chief

Increasing the awareness and knowledge of breast cancer (BC) is the most direct way to improve women’s behavior for participating prevention programs.

In this cross-sectional study, the awareness of BC was investigated in patients and relatives in a Breast-Endocrine Clinic, Ankara Numune Training and Research Hospital, Turkey between May and July 2016. The survey form was modified from a study on BC awareness of hospital staffs (1).

The participants were informed about the purpose of the study and verbal informed consent was obtained from all.

Among 164 participants, 75% of the participants were female, and 54.8% were under 45. Educational status was primary/secondary school (53.7%) and ≥ high school (46.3%). The majority of participants (82.9%) had no family history of BC. Age, family history, hormone replacement treatment (HRT), and early menarche were identified as a risk factor of BC by approximately 75%, 60%, 50%, and 35% of the participants, respectively. The overall success of general belief section was 80%. The comparison of answers according to age, gender, and educational status is presented in Table 1.

**Table 1:** Comparison of answers about BC

	***Correct n (%)***	***Wrong n (%)***	***P***	***Correct n (%)***	***Wrong n (%)***	***P***
	***Does age increase BC risk?***			***Is BC a contagious disease?***		
Gender						
Male	26 (76.4)	8 (13.6)	0.748	32 (100)	0 (0)	
Female	90 (72.5)	34 (26.5)		86 (74.1)	30 (25.9)	**0.031**
Education						
Primary/Secondary	62 (73.8)	22 (26.2)	0.933	58 (70.7)	24 (29.6)	
≥High school	54 (84.4)	10 (15.6)		60 (90.9)	6 (9.1)	**0.032**
Age						
>45	53 (63.1)	31 (36.9)	**0.048**	54 (73)	20 (27)	0.248
≤45	62 (83.8)	12 (16.2)		59 (82)	13 (18)	
	***Does HRT increase BC risk?***			***Is BC a fatal disease?***		
Gender						
Male	10 (29.4)	24 (70.6)		22 (64.7)	12 (35.3)	
Female	66 (56.9)	50 (43.1)	**0.046**	66 (56.4)	51 (43.6)	0.688
Education						
Primary/Secondary	44 (53.6)	38 (46.4)		50 (61)	32 (39)	
≥High school	36 (53)	32 (47)	0.569	36 (54.5)	30 (45.5)	0.577
Age						
>45	44 (57.9)	32 (42.1)		40 (54.1)	34 (45.9)	
≤45	32 (42.1)	42 (57.9)	0.204	46 (62.1)	28 (37.9)	0.480
	***Does family history of BC increase BC risk?***			***Do only females have BC?***		
Gender						
Male	18 (56.2)	14 (43.7)	0.768	24 (75)	8 (25)	
Female	72 (60)	48 (40)		68 (58.6)	48 (41.4)	0.232
Education						
Primary/Secondary	48 (58.5)	34 (41.5)	0.897	42 (48.8)	44 (51.2)	
≥High school	42(60)	28 (40)		52 (78.8)	14 (21.2)	**0.008**
Age						
>45	46 (58.9)	32 (41.1)	0.966	44 (59.4)	30 (40.6)	
≤45	44 (61.1)	28 (38.9)		48 (64.9)	26 (35.1)	0.632
	***Does early menarche increase BC risk?***					
Gender						
Male	4 (12.5)	28 (87.5)	**0.024**			
Female	50 (43.1)	66 (56.9)				
Education						
Primary/Secondary	32 (39)	50 (61)	0.613			
≥High school	22 (33.3)	44 (66.7)				
Age						
>45	30 (40.5)	44 (59.5)	0.469			
≤45	24 (32.4)	50 (67.6)				

* Chi-square/Fisher exact tests were used for statistical analyses.

** *P*<0.05 (significance level)

The overall awareness about BC risk factors in our study was 50%, similar to the literature (2). Age and family history were the best-known risk factors. In general, belief section, correct answer rate was higher than that in other sections. The overall awareness was better than normal population in our study. In our opinion, the overall high correct answer rate of participants pointed out that exposure to the disease forced responders to do more research on BC.

Awareness of BC tends to decrease with increasing age (3). In our study, subgroup analysis about age failed to reveal a decrease, probably based on increased awareness of older responders due to the reason for doing more research BC. Gender is also a determining factor for awareness of cancers. In a study, male participants had similar knowledge about “uni-sex” cancers such as lung, but for BC, the level of awareness was determined significantly lower when age and education adjustments were made (4). In our study, questions about HRT and early menarche had a correct answer rate in females. This can be explained by the opinion that risk factors defined as “complex” seem to be more familiar with women. Educational status is also related to awareness. In our study, well-educated responders had a correct response rate of 80% higher than the literature (5). BC symptoms were often well known by our participants. As expected, the best-known symptoms were mass and change in breast shape. The weight of the correct answer from female participants also draws attention in this section.

Well-educated patients with a breast disease or their relatives can be a correct source for education of general population. Initiation of the education programs for this group may be a feasible option to realize this idea.
